# Physicochemical and Rheological Characteristics of Monofloral Honeys—Kinetics of Creaming–Crystallization

**DOI:** 10.3390/foods14101835

**Published:** 2025-05-21

**Authors:** Kerasia Polatidou, Chrysanthi Nouska, Chrysoula Tananaki, Costas G. Biliaderis, Athina Lazaridou

**Affiliations:** 1 Laboratory of Food Chemistry and Biochemistry, Department of Food Science and Technology, School of Agriculture, Aristotle University of Thessaloniki, P.O. Box 235, 54124 Thessaloniki, Greece; kerypolatidou@hotmail.com (K.P.); cinouska@agro.auth.gr (C.N.); biliader@agro.auth.gr (C.G.B.); 2 Laboratory of Apiculture–Sericulture, School of Agriculture, Aristotle University of Thessaloniki, 54124 Thessaloniki, Greece; tananaki@agro.auth.gr

**Keywords:** sugar content, steady shear rheological measurements, oscillatory rheological measurements, glass transition temperature, Gordon–Taylor model, honey crystallization, creaming kinetics, power-law fitting

## Abstract

The quality and stability of honeys are strongly influenced by their chemical composition and physicochemical properties, which vary with botanical origin. This study examined the physicochemical and compositional properties of cotton, heather, orange, thyme, Christ’s thorn, and chestnut monofloral honey samples, as well as the kinetics of the creaming–crystallization process by monitoring rheological and color parameters. All samples had moisture content lower than the legislation limit (<20%) and a_w_ ≤ 0.60. Chestnut and heather honeys exhibited the highest electrical conductivity and darkest color. Fructose was the predominant sugar in all samples, with thyme having the highest content. Viscosity decreased exponentially with increasing moisture, with thyme honey being the most viscous. Principal component analysis showed distinct clustering of samples based on their compositional–physicochemical characteristics. Calorimetry revealed the water’s plasticization effect on honey solids, lowering their glass transition temperature, with the data fitting well to the Gordon–Taylor model. Rheometry indicated a Newtonian-like behavior for liquid honeys, evolving towards a pseudoplastic response upon creaming–crystallization. Cotton honey crystallized rapidly, thyme honey showed moderate crystallization propensity, while samples of heather honey gave a diverse response depending on composition. Overall, high glucose content and/or low fructose/glucose ratio promoted honey crystallization, leading to the formation of highly viscous-creamed honey preparations.

## 1. Introduction

Honey is predominantly composed of the monosaccharides glucose and fructose, along with other sugars present in lower concentrations, as it is characterized by its supersaturated nature (~95% of honey’s dry matter is sugars) [1,2]. Fructose generally constitutes the primary sugar in honeys, although for certain species, such as dandelion (*Taraxacum officinale*) and rape (*Brassica napus*) honey, glucose is the dominant sugar component [3]. Besides sugars, honey contains low concentrations of proteins, minerals, organic acids, flavor and aroma compounds, pigments, colloidal particles, vitamins, sugar alcohols, and other phytochemicals, all of which contribute to its physicochemical and bioactivity aspects [4]; for the latter, antioxidant, antiviral, antibacterial, antifungal, antitumor, antidiabetic, anti-inflammatory and anticancer properties of honey are generally attributed to its minor components [5,6,7]. The chemical composition of honey is greatly influenced by geographical origin, climatic conditions, and plant botanical sources [8], whereas its physicochemical properties are affected by the floral origin, as these characteristics are directly linked to plant-derived secretions, nectar, and honeydews [9].

Crystallization is a natural process that occurs in honey that is primarily driven by formation–precipitation of glucose as crystalline entities of glucose monohydrate [10,11,12]. While some honeys crystallize rapidly, others exhibit slower rates of crystallization due to differences in sugar composition, moisture content, and viscosity [13]. An increase in viscosity delays crystallization, whereas higher supersaturation of sugars accelerates the process. Maximum crystallization is typically observed at temperatures between 10 and 15 °C [2,4]. Temperatures exceeding 18 °C reduce the rate of crystallization due to decreased glucose supersaturation, while at temperatures above 30 °C, sugar crystals undergo transition to a liquid state (solubilization). Conversely, at temperatures below 10 °C, a slow crystallization rate occurs due to the increased viscosity and reduced molecular mobility, resulting in the formation of smaller crystals [14,15].

To predict honey crystallization, the fructose-to-glucose (F/G) ratio is widely accepted as a molecular marker for crystallization propensity; specifically, honeys are categorized as slow crystallizing (F/G > 1.33), medium crystallizing (1.11 ≤ F/G ≤ 1.33), and rapid crystallizing (F/G < 1.11) [16]. Another important indicator is the glucose-to-water (G/W) ratio, where values exceeding 2.0 correspond to rapid and complete crystallization, while values below 1.7 imply slow crystallization [17,18]. A combination of a low F/G ratio and a high G/W ratio typically leads to rapid crystallization [19]. Water content also affects the crystallization process, as higher water levels increase glucose solubility and reduce viscosity, thereby lowering the crystallization rate [11].

Furthermore, storage conditions significantly influence the crystallization process and the resulting crystal characteristics. At −20 °C, honey forms fine crystals, while at 20 °C, coarse crystals develop. In the intermediate range of 4–10 °C, mixed-size crystals occur due to the reduced molecular mobility at lower temperatures, which promotes nucleation and formation of numerous crystals of smaller size [20]. Although crystallization does not alter the nutritional or chemical characteristics of honey [21], it is often perceived as an undesirable feature by consumers due to its cloudy appearance and unpleasant organoleptic sensation (grainy texture) caused by unrestrained crystallization upon storage [11]. Moreover, upon crystallization of glucose, there is an increased amount of free water, resulting in higher water activity (a_w_) values, thereby allowing microbial growth (osmophilic yeasts).

Controlled crystallization processes are being adopted to address the above concerns, yielding a more uniform product with improved sensory attributes, extended shelf-life [22], enhanced physical properties, and better spreadability [21]. Such products, referred to as ‘creamed honey’, are highly valued for their smooth texture and uniform consistency. In contrast, uncontrolled crystallization often results in coarse and grainy textures that are less appealing to consumers (visually and mouthfeel perception) due to distinct phase separation of coarse crystals, being dispersed in the remaining liquid sugar medium [23].

Heating is another factor that influences honey quality. While high-temperature treatments can control crystallization by eliminating small sugar nuclei and other particles upon which sugar crystallization can be built on their surfaces on storage (heterogeneous nucleation), they may negatively impact diastase activity, an enzyme introduced by bees during nectar processing. Moreover, heating can lead to an increase in hydroxymethylfurfural (HMF) formation [24]. Therefore, both diastase activity and HMF concentration are being used as indicators of honey thermal processing under harsh conditions. Changes in honey composition upon heating underscore the importance of balancing processing conditions to preserve sensorial and bioactive properties of honey products.

Overall, honey’s quality is assessed by a range of parameters, including fructose, glucose and sucrose contents, water content, dispersed insoluble matter, HMF, diastase activity, free acids, electrical conductivity, sensory attributes, and microbiological factors [25,26].

The objective of this study was to investigate the influence of botanical origin on the compositional, physicochemical, and rheological characteristics of Greek monofloral honeys derived from six plant sources: thyme, Christ’s thorn, cotton, heather, orange, and chestnut. The estimated parameters included moisture content, water activity (a_w_), electrical conductivity, HMF, diastase activity, color, sugar composition, viscosity, and glass transition temperature, as well as possible correlations between these variables were sought. Additionally, the crystallization rate, and changes in the rheological behavior, and color lightness of representative thyme, cotton, and heather honey samples were examined throughout a creaming process to offer practical insights for handling and storage of such monofloral honeys; in this context, cotton, heather, and thyme honeys were selected according to their diversity in compositional characteristics and crystallization indices. This work follows up on our previous study [23], which evaluated the physical properties of a formulated cotton honey-based spread product made by a creaming process, without examining the kinetics of structure formation or the effects of the honey’s botanical origin. By addressing the above aspects, the study aims to contribute to a broader understanding of honey quality, while providing producers, quality control laboratories, and regulators with practical indicators to support improved processing, product quality assurance, and overall consumer appeal.

## 2. Materials and Methods

### 2.1. Raw Materials

Honey samples were collected in Greece from the Laboratory of Apiculture–Sericulture, of Aristotle University of Thessaloniki. These samples were stored at 20 °C and represented a range of monofloral honeys that meet the Greek legislated requirements for pollen composition (type and content). Specifically, we collected five samples of thyme (*Thymus vulgaris*) honey, having thyme pollen content > 18%, five samples of Christ’s thorn (*Paliurus spina-christi*) honey with C. thorn pollen content > 45%, three samples of orange (*Citrus sinensis*) honey with orange pollen content > 3%, four samples of chestnut (*Castanea sativa*) honey with chestnut pollen content > 87%, three samples of heather (*Erica manipuliflora*) honey with heather pollen content > 45%, and two samples of cotton (*Gossypium* spp.) honey with cotton pollen content > 3%.

### 2.2. Preparation of Samples

Liquefaction (crystal melting) was initially carried out on samples by heating them to 45 °C. A sample was considered liquefied when the crystal content was consistently below 0.1%, and no crystals could be observed microscopically. Portions of 150 g of honey from each liquefied sample were transferred into glass jars and stored in a freezer until analysis.

For the production of creamed honey, three different monofloral type of honeys were used: thyme, cotton, and heather. For each sample, 100 g of honey was transferred into glass jars with screw caps, and 10 g of creamed honey was added as a crystallization starter (seeding). The mixture was then stirred at the lowest speed for 15 min using a mixer (KENWOOD, KM 282, Havant, UK). The samples were subsequently stored at a constant temperature of 14 °C for 27 d. Rheological analysis and colorimetry were performed every two days.

### 2.3. Physicochemical Parameters of Honey

#### 2.3.1. Moisture Content

A hand-held honey moisture refractometer (ATAGO, HHR-2 N, Tokyo, Japan) was used at 20 °C with a precision level of 0.1% to estimate the moisture content of the samples.

#### 2.3.2. Water Activity

Water activity (a_w_) of the samples was measured using a water activity meter at 20 °C (Aqualab 3TE, Decagon Devices, Inc., Pullman, WA, USA).

#### 2.3.3. Electrical Conductivity

Determination of electrical conductivity, related to the mineral and organic acid composition of honey, was carried out at 20 °C, following the method of IHC (International Honey Commission) and using a certain amount of honey sample dissolved in distilled water [27]. The electrode of the electrical conductivity meter (WTW COND 315i/set, WTWGmbH, Stuttgart, Germany) was immersed in the aqueous honey solution, and the conductivity was expressed in mS·cm^−1^.

#### 2.3.4. Color

The color of the honey samples was evaluated using a chromameter (Konica Minolta CR-410, Konica Minolta Inc., Wayne, NJ, USA) to measure the CIE L* (lightness, 0–100), a* (positive range: red; negative range: green) and b* (positive range: yellow; negative range: blue) values. Color measurements of the creamed honey samples were conducted every two days over a 27-day storage period.

#### 2.3.5. Hydroxymethylfurfural Analysis (HMF)

- Sample preparation:

A portion of 5 g of honey was dissolved in distilled water and the volume adjusted to 25 mL. Subsequently, 0.5 mL of Carrez I solution (15% *w*/*v* K_4_Fe(CN)_6_·3H_2_O, Merck KGaA, Darmstadt, Germany) and 0.5 mL of Carrez II solution (30% *w*/*v* Zn(CH_3_CO_2_)_2_·2H_2_O, Merck KGaA, Darmstadt, Germany) were added and the final volume was adjusted to 50 mL with distilled water. The solution was filtered (Whatman^®^ qualitative filter paper (Maidstone, UK), Grade 1), and the filtrate was collected, ensuring that the first 10 mL was discarded.

- Spectrophotometric analysis:

Portions of 5 mL of the collected filtrate were transferred into test tubes. For the blank (reference), 5 mL of NaHSO_3_ (0.2% *w*/*v*) were added, whereas in the case of the sample, 5 mL distilled water was added. The mixtures in the tubes were then vortexed, and the absorbance was measured at 284 and 336 nm with a UV-1800 spectrophotometer (Shimadzu, Kyoto, Japan). The HMF concentration was calculated in mg/kg according to the following equation [28]:


HMF (mg/kg) = (A284 − A336) × 149.7
(1)

#### 2.3.6. Diastase Activity

- Starch solution titration procedure:

An aliquot of 5 mL of starch solution (1% *w*/*v*) was mixed with 10 mL of distilled water. 1 mL of this diluted starch dispersion and 10 mL of dilute iodine solution (0.0007 N) were mixed with 35 mL of distilled water. The absorbance was expected to be approximately 0.760 ± 0.020 at 660 nm. If the absorbance was out of this range, the water content was adjusted accordingly to achieve the desired absorbance.

- Honey analysis:

A portion of 10 g of honey was dissolved in 20 mL of distilled water and 5 mL of the acetate buffer (1.59 M, pH 6.0) at room temperature. A portion of 3 mL of a sodium chloride solution (0.5 M) was then added, and the final volume was adjusted to 50 mL. Aliquots of 10 mL of the honey solution were stored at 40 °C, and mixed with 5 mL of starch solution and at each time point 1 mL of this mixture was further diluted with the previously mentioned water and iodine solution (0.0007 N), and the absorbance at 660 nm was measured [29].

For calculation of diastase activity, a least-squares regression line was plotted with time (min) on the *x*-axis and absorbance at *y*-axis. The time corresponding to an absorbance of 0.235 was obtained from the equation. Diastase activity, expressed as diastase number (DN), was calculated by dividing 300 by the determined time.

#### 2.3.7. Sugar Composition

Sugar analysis was performed using High Performance Liquid Chromatography (HPLC, Agilent Technologies 1200, Tokyo, Japan), according to the method outlined by the International Honey Commission [27]. Briefly, 1 g of the sample was dissolved in 10 mL of a methanol/water (25:75 *v*/*v*) mixture (Chem-Lab NV, Zedelgem, Belgium). After filtration through a 0.45 μm nylon filter (BGB Analytik USA LLC, Alexandria, VA, USA), 10 μL of the sample was injected into the HPLC system. The separation was carried out isocratically using a Zorbax Carbohydrate Analysis Column (4.6 mm ID × 150 mm × 5 μm). A refractive index detector was used (Agilent 1200 series, Santa Clara, CA, USA), and the mobile phase consisted of acetonitrile/water (80:20 *v*/*v*, Sigma-Aldrich, St. Louis, MO, USA) with a flow rate of 1.3 mL/min at 30 °C. For quantitative determination, five-point calibration curves were constructed for each carbohydrate component.

### 2.4. Rheological Properties

The rheological properties of the honey samples were measured using a rotational Physica MCR 300 rheometer (Physica Messtechnic CmbH, Stuttgart, Germany). For the honey samples, a plate–plate geometry was used (50 mm diameter), and the gap between the plates was set at 1 mm. To regulate the temperature, a Paar Physica circulating bath and a controlled peltier system (TEZ 150P/MCR) were used.

To assess the flow behavior of the honey samples, steady shear viscosity (η) and shear stress (σ) were measured over a range of shear rates ($\dot{\gamma \;}$) of 0.01–200 s^−1^ at 20 °C. Moreover, oscillatory measurements were conducted to determine the storage (G′) and loss (G″) moduli, as well as the complex viscosity (η*), over a range of angular frequencies (ω) of 0.1–100 Hz at 20 °C and 0.1% strain. For the study of creaming process kinetics, the rheological testing of honey samples was performed every two days over a 27-day period.

Moreover, the power-law model was used to describe the relationship between shear rate and shear stress in creamed honey samples during storage, using Equation (2) [30].

(2) $$\sigma =K\text{ ×}{\dot{\;\gamma \;}}^{n}$$

where σ is the shear stress, K is the consistency coefficient (Pa∙s^n^), and n is the flow behavior index.

### 2.5. Thermal Analysis

Differential Scanning Calorimetry (DSC) was conducted using a PL DSC-Gold calorimeter (Polymer Labs Ltd., Epsom, UK). Samples weighing 68.9–95.4 mg were hermetically sealed in aluminum pans, with an empty pan used as the reference. The temperature range was from 20 °C to −75 °C (using liquid nitrogen), followed by reheating to 20 °C at a heating rate of 10 °C/min. The glass transition temperature (Tg) was determined from the second thermal scan as the onset temperature of the vertical shift in the heat flow curve. The Gordon–Taylor empirical equation [31] was applied by fitting the Tg data to the water weight fraction of the honey samples according to the following equation:

Tg = (w_1_∙Tg_1_ + k∙w_2_∙Tg_2_)/(w_1_ + k∙w_2_) (3)

where w_1_ and w_2_ are the weight fractions of honey and water, respectively, Tg_1_ is the Tg of the dry honey samples, Tg_2_ is the Tg of amorphous water (−138 °C) and k is a constant related to the strength of matrix-diluent interactions (the larger the k, the greater the plasticization effect). Parameters k and Tg_1_ were estimated by fitting the experimental data to the mathematical model, using the nonlinear regression of the Excel Solver tool (Excel, Microsoft 365, Microsoft Corporation, Redmond, WA, USA).

### 2.6. Statistical Analysis

All experimental values were expressed as means ± standard deviations based on at least three measurements per honey sample. Subsequently, the results of the honey samples were summarized into a single value. Analysis of Variance (ANOVA) was conducted for all experimental data, followed by Tukey’s test at a 95% significance level. Pearson correlations were also conducted to examine the relationships among the rheological parameters (G′, G″, η*, η, K, n) and color parameter (L*), at the last day of the creaming process, and the compositional parameters (F/G ratio, glucose) of the honey samples. Both types of statistical analysis were carried out by the IBM SPSS statistical software (version 22.0, IBM Corp., Armonk, NY, USA).

Moisture content, a_w_, diastase activity, HMF content, G′ and G″ moduli (at 1 Hz), L* color parameter, fructose and glucose content and fructose/glucose (F/G), fructose+glucose (F + G) and glucose/water (G/W) values were interrelated using Principal Component Analysis (PCA) by employing the Minitab version 18 software (Minitab, Inc., State College, PA, USA).

## 3. Results and Discussion

### 3.1. Physicochemical Parameters of Honeys

#### 3.1.1. Moisture Content

The mean values of moisture content of monofloral honeys ranged from 14.60% to 18.10% (Table 1). All tested samples exhibited moisture content below 20%, which is the maximum limit for composition-stability, according to the Council of the European Union [32]. High moisture levels were observed for heather honeys and low for thyme honeys. A low moisture content can protect honey from microbial spoilage [33].

#### 3.1.2. Water Activity

The highest mean water activity value was observed for heather honey (0.60), whereas thyme honey exhibited the lowest water activity (0.53) (Table 1), consistent with the trend observed for moisture content of the tested samples. High moisture content is correlated with increased water activity, which facilitates the growth of osmophilic yeasts, causing fermentation–spoilage of the product. Moreover, crystallization is often accelerated (due to reduced viscosity), with further increase of free (unbound) water, which results in additional increase of the a_w_ and, thus, the sensory attributes of honey are largely altered. However, all samples exhibited a_w_ values ≤ 0.60, suggesting that these honeys are microbiologically stable. Overall, the low water activity implies low free water content, reducing the potential for chemical reactions, microbial growth and enzymatic activity [34].

#### 3.1.3. Electrical Conductivity

Chestnut and heather honeys exhibited the highest electrical conductivity (Table 1), while thyme and orange honeys demonstrated the lowest values. The electrical conductivity of honey is influenced by the floral origin of the sample and the concentration of proteins, organic acids, minerals, and ions. Therefore, electrical conductivity can serve as a reliable index for differentiating honeys from various floral sources [35,36]. Moreover, high electrical conductivity values are indicative of higher mineral content, reflecting the richness of the honey’s composition.

Electrical conductivity is also useful in distinguishing between honeydew and blossom honeys, with honeydew honeys typically displaying higher conductivity values than honeys produced from nectar [37]. However, some floral honeys, such as heather, can show high electrical conductivity despite being derived from nectar [38]. According to the Council of the European Union, nectar honeys generally exhibit conductivities below 0.80 mS/cm, with a few exceptions, whereas honeydew honeys tend to exceed 0.80 mS/cm. Thyme and orange honey samples conformed to the legislation, in contrast to C. thorn, chestnut, and heather honey, which surpassed the recommended range of electrical conductivity value, suggesting a higher mineral content [39,40]. Variations in electrical conductivity of honey samples are known to be strongly linked to the botanical origin of the product [41,42].

#### 3.1.4. Color

The color characteristics of honey (Table 1) are strongly influenced by its botanical origin and chemical composition. Cotton honey samples exhibited the highest mean value for L* parameter, followed by orange and thyme, reflecting a light color intensity, whereas chestnut and heather honeys showed the lowest L* values (Table 1), indicative of their darker appearance. This variation reflects the impact of plant origin in determining the darkness of honey samples.

In terms of the a* parameter, which measures red–green coloration, chestnut, C. thorn, and heather honeys displayed the highest values, followed by thyme, cotton, and orange honeys. Moreover, cotton and thyme honeys demonstrated high b* values, while chestnut and heather samples exhibited the lowest. These findings highlight the complex interplay between honey’s botanical origin and its color properties, which may also reflect the formation of colored complexes between organic compounds and transition elements [43].

Honey color is also influenced by its content of phenolic compounds, including flavonoids, and minerals [44], as darker honeys have been shown to correlate with higher concentrations of flavonoids and polyphenolic compounds [45]. The predominant sugars in honey, such as monosaccharides, may further contribute to color development, as they can participate in Maillard reactions during processing and/or storage, particularly if thermal treatments are applied to the product [46]. Differences in color among honey samples can additionally arise from external factors, including beekeeper practices, production methods, storage conditions, exposure to light, contact with metals, and elevated temperatures [47].

#### 3.1.5. Hydroxymethylfurfural Content (HMF)

Heather honey exhibited the highest mean values of HMF content (Table 2), while the lowest values were noted for chestnut samples. HMF content serves as an indicator of honey quality, reflecting the degree of deterioration and freshness [48]. Generally, the highest HMF content recorded was 37.1 ± 1.2 mg/kg in a heather honey sample, which is however still below the upper regulation limit of 40 mg/kg set by the European Commission [32]. This suggests that all tested samples were fresh and were not overheated, since heating processes are known to enhance HMF formation [24].

#### 3.1.6. Diastase Activity

The highest mean value of diastase activity was observed in C. thorn honey, followed by chestnut, cotton, thyme, and orange honey, while the lowest mean diastase activity was noted for heather honey (Table 2). Moreover, all mean diastase activity values exceeded the minimum regulatory limit of 8 DN [32]. However, some samples of thyme, heather, and orange honey showed diastase activity values below the regulation limit of 8 DN. Diastase enzyme (amylase) is involved to the conversion of starch to maltose and glucose; it is transmitted by bees’ saliva during nectar processing and serves as an indicator of enzymatic activity presence in honey. Heating processes can decrease diastase activity [24], which makes this parameter significant for assessing honey quality.

Both diastase activity and HMF content are widely recognized as indicators of honey quality, particularly for honeys that have been subjected to prolonged high-temperature processing or are not fresh [49]. Furthermore, storage conditions can impact diastase activity, as enzymatic inactivation may also occur upon prolonged storage [50]. Consequently, honeys exhibiting lower HMF content and higher diastase activity are generally considered to be fresher and less overheated [48].

#### 3.1.7. Sugar Composition

The sugar composition of all tested honey samples is presented in Table 3. Thyme honey had the highest mean fructose content (40.75 ± 5.18 g/100 g), while the lowest was observed in heather honey (32.46 ± 5.12 g/100 g). All the other tested samples had similar fructose content. The mean glucose content was higher in cotton honey (38.42 ± 2.33 g/100 g) and lower in C. thorn and chestnut honeys (24.72 ± 2.13 and 27.73 ± 2.94 g/100 g, respectively). In all samples, fructose dominated over glucose, while all the other sugars were found at lower concentrations. Overall, fructose and glucose were the major sugar constituents, as previously reported [51,52,53]; the total content of the predominant invert sugars (fructose+glucose) was higher in thyme and cotton honeys and lower in C. thorn honey. The sum of these sugars is an index to evaluate the origin of honeys [18]. Blossom honeys must have invert sugar content higher than 60 g/100 g, whereas honeydew honeys have higher than 45 g/100 g [54]; the respective levels were exceeded for all tested honeys, except some samples of C. thorn.

Honey is a liquid food which may undergo crystallization upon storage. Honeys with high glucose content typically crystallize more rapidly. As a physicochemical process, honey granulation may be explained by the lower solubility of glucose compared to fructose; thus, the ratio of fructose to glucose (F/G) serves as a valuable index for predicting crystallization potency [55]. Ideally, the F/G ratio falls between 0.9 and 1.35, with values below 1.0 indicating rapid crystallization and values above 1.0 suggesting slower crystallization [56]. All tested samples exhibited a F/G ratio higher than 1.0, suggesting slow crystallization [18,57,58]. Cotton honey presented the lowest F/G value (1.01), suggesting faster crystallization than C. thorn honey (1.38), which had the highest F/G ratio.

Additionally, crystallization is influenced by both water and sugar content, with the ratio of glucose to water (G/W) representing another crystallization index [57]. Cotton honey exhibited the highest mean value of G/W ratio (2.43), while the lowest ratios were observed for heather (1.64) and C. thorn honeys (1.54). Considering all these crystallization indices (F + G, F/G, and G/W), it is expected that cotton honey will crystallize faster than the other tested honeys.

The third most abundant sugar was maltose, which can also influence the crystallization process, but only when present at high levels, such as in cases of adulteration (e.g., with starch hydrolysates), resulting in slower crystallization [59]. However, all tested samples showed low maltose content.

Sucrose was present only in thyme and orange honeys. Higher sucrose content can lead to faster crystallization and affect the consistency and texture of honey [60]. Sucrose levels should be less than 5 g/100 g, as higher amounts may indicate honey adulteration with this sugar or implies that the bees were fed with sugar syrups, a practice associated with food fraud [57] or that honey was not fully ‘ripened’ when harvested, and the invertase enzyme in the bees was not able to fully hydrolyze sucrose into fructose and glucose [51]. Nevertheless, both thyme and orange honeys had sucrose content below this threshold.

Trehalose, a non-reducing disaccharide of α-D-glucose, was found in low levels in all samples, while raffinose was detected only in thyme honey. Melibiose, a reducing disaccharide between galactose and glucose interlinked by an α-1,6 linkage [D-Gal-(α1→ 6)-D-Glc] was also detected in some samples. Melezitose (a non-reducing trisaccharide), which indicates the presence of honeydew [55,61], was not found in any of the tested samples.

### 3.2. Rheological Behavior

**Figure 1:**
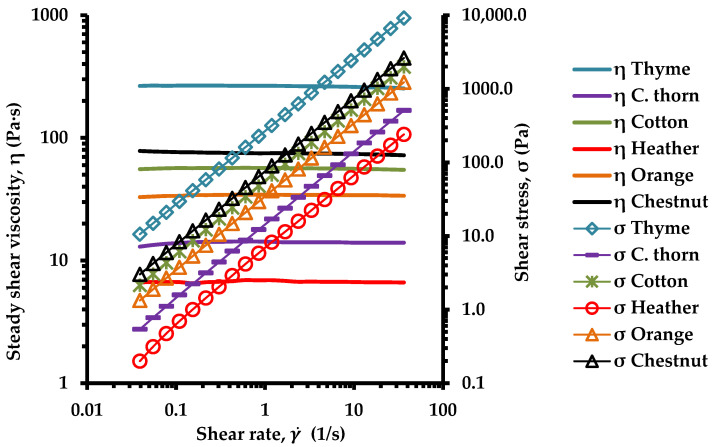
Flow curves of six representative Greek honey samples from six plant origins; measurements were performed at 20 °C.

**Figure 2:**
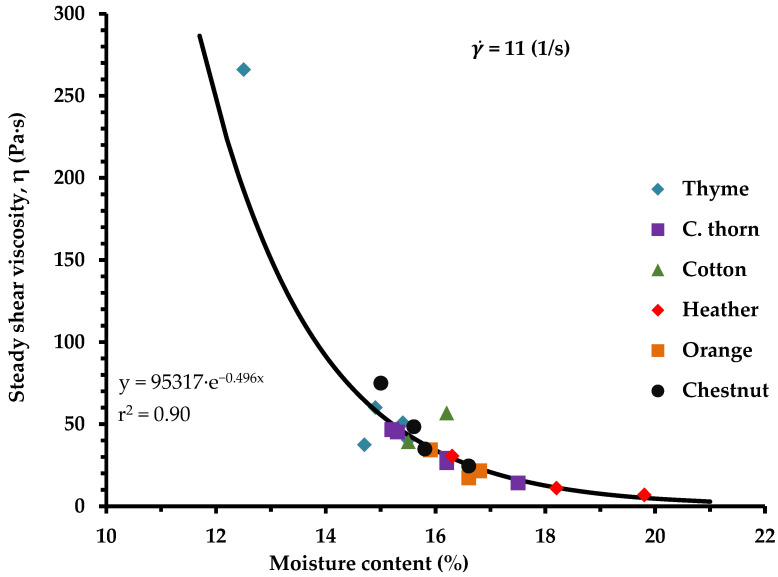
Correlation of moisture content and steady shear viscosity (at 20 °C) of Greek honey samples from six plant origins.

All liquified honey samples exhibited Newtonian behavior, based on the apparent viscosity (η) values which were independent of shear rate ($\dot{\gamma \;}$), and the shear stress (σ) linearly varied with the shear rate, on a log–log scale (Figure 1). Many previous researchers have also noticed Newtonian behavior for liquid honeys [62,63]. Thyme honey exhibited high viscosity (η) values, while the heather honey samples displayed low viscosity (Figure 1 and Figure 2). These differences are likely attributable to the distinct botanical origin of the honey samples which can lead to compositional differences; previous studies have suggested that small variations in the water content and sugar composition of honey can influence these rheological properties [64,65,66].

Accordingly, heather honey, which had the highest moisture content of the tested samples, exhibited the lowest viscosity, whereas thyme honey, exhibited the lowest moisture content (Table 1) and a high sugar content level (Table 3), and displayed the highest viscosity (Figure 1 and Figure 2). Therefore, a strong relationship was found between the steady shear viscosity, η, and moisture content of the honey samples, implying that the decrease in moisture content results in an exponential increase of honey viscosity (Figure 2), as has been suggested by previous studies as well [66,67,68].

### 3.3. Thermal Behavior

**Figure 3:**
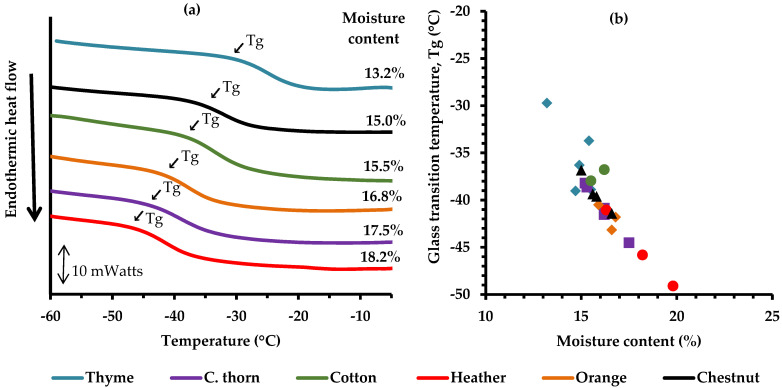
Representative thermal scans of Greek monofloral honey samples with different moisture content (**a**) and effect of moisture content on glass transition temperature (Tg) (**b**); the Tg values were estimated by differential scanning calorimetry from the onset of heat flow drop in the second thermal scan (10 °C/min heating rate).

The glass transition temperature (Tg) of honey samples ranged from −29.72 °C for thyme honey to −49.09 °C for heather honey, within a moisture content range of 13.2% to 19.8% (Figure 3). Overall, the composition of honey is a major determinant of its Tg value, as samples containing low molecular weight compounds (e.g., sugars) exhibit lower Tg values. Since honey primarily consists of fructose and glucose, both with low molecular weights (~180 g/mol) and low Tg values (~5 °C and 31 °C, in their dry state, respectively) [69], the Tg of honey is expected to be low and it further decreases with the presence of small amounts of water (effective plasticizer for hydrophilic solutes, such as sugars). Apparently, a small increase in moisture content can lead to an abrupt decline of the Tg (Figure 3), a phenomenon attributed to the plasticizing action of water on honey solids [66,70]. Since thyme honey had the lowest moisture content (Table 1), it exhibited the highest Tg among the samples, while heather exhibited the lowest due to its highest moisture content among the other species (Table 1, Figure 3).

The glass transition temperature region represents the point where state transitions of hydrated honey solids occur, i.e., from a glassy to a rubbery state [71,72]. Several factors, including crystallization, water activity, and moisture content, are known to influence Tg [70]. In addition to moisture content (major impact), the type of solute (sugar) could affect the Tg values of sugar solutions [14]; however, such effect was not noticed for honey samples examined in the present study, possibly because the differences in the sugar composition among the tested samples were not large enough to have a measurable impact on Tg values as determined by calorimetry.

**Figure 4:**
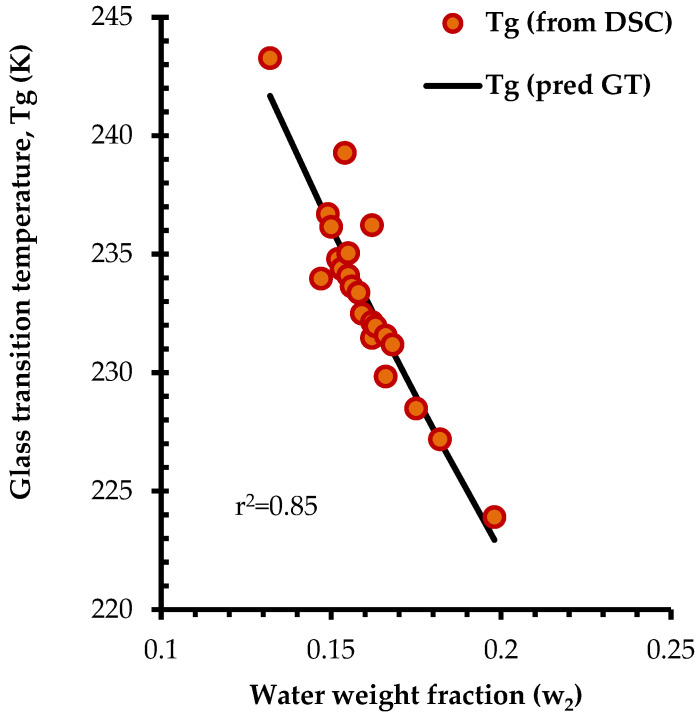
Fitting of the experimental glass transition temperature (from differential scanning calorimetry, DSC) of honey samples from different origin versus the water weight fraction (w_2_) to the Gordon–Taylor (GT) equation (predicted GT line is also shown); experimental Tg values were estimated by DSC from the onset of heat flow drop in the second thermal scan (10 °C/min heating rate).

The inverse relationship between moisture content and Tg, as illustrated in Figure 3b, reveals the strong plasticization effect of water on honey solids. Regardless of botanical origin, the honey samples followed the same trend, with higher moisture content resulting in lower Tg values. This effect is explained by the presence of hydrophilic compounds (sugars exhibiting compatibility with water upon hydration), of small molecular weight (sugars), and increased free volume, all of which enhance hygroscopicity and lead to lower Tg values [73]. To further explore the relationship between moisture and Tg of the liquid honeys, the experimental data were fitted to the Gordon–Taylor (GT) equation (Figure 4). The data fitting was reasonably good (r^2^ = 0.85) and the estimated values of k and Tg_1_ from the GT model were 3.41 and 24.05 °C (predicted Tg value of dry honey), which are in close agreement with previous findings [66].

### 3.4. Principal Component Analysis

**Figure 5:**
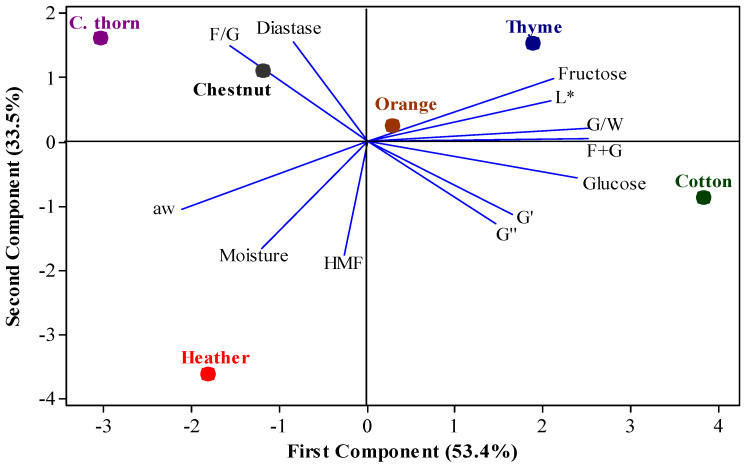
Principal Component Analysis (PCA) of liquified honeys from different plant origin. L*, color parameter; G/W, glucose/water ratio; F+G, sum of fructose and glucose; G′, storage modulus at 1 Hz; G″, loss modulus at 1 Hz; HMF, hydroxymethylfurfural; aw, water activity; F/G, Fructose/glucose ratio.

Figure 5 presents the Principal Component Analysis (PCA) scores for the examined compositional, physicochemical, and rheological parameters of the honey samples. The first principal component accounts for 53.4% of the variance, while the second component explains 33.5%, resulting in a total variance of 86.9%. Honeys originating from different plant species are grouped into distinct quadrants, reflecting their differences in the examined parameters. Cotton honeys appeared to be associated with light color (high L* parameter), high glucose content, and high viscoelastic moduli values (G′ and G″), while heather honeys were distinguished for their high moisture content, a_w_ and HMF values.

Notably, C. thorn and chestnut honeys appear in the same quadrant, suggesting they are distinguished by the same characteristics, which were the high diastase activity and F/G ratio. Similarly, thyme and orange honeys are clustered together based on their high L* parameter, fructose content, G/W ratio and sum of fructose and glucose. However, thyme, orange, and cotton honey seemed also to be associated with the same characteristics (strong positive correlations with L*, fructose, glucose, F+G and G/W) based on the small angles between the vectors of these variables for the above honey groups. Overall, the PCA results underscore the significant impact of botanical origin to the relationships among the tested parameters.

Furthermore, a strong positive correlation is noted between the F/G ratio and diastase activity, while a negative correlation is noted between these parameters and G′, G″ and glucose content. Additionally, the fructose content, the L* color parameter, the ratio of G/W and the sum of fructose and glucose (F + G) exhibited a strong positive correlation, whereas negative correlations of these parameters were observed with moisture content, water activity (a_w_), and HMF levels. Negative correlation between HMF and the L* color parameter has been postulated in earlier studies [74,75]. Moreover, previously observed interrelations between fructose and glucose content, F/G ratio, moisture content, and G″ of honey samples [76] are consistent with our findings.

### 3.5. Crystallization Kinetics of Creaming Process of Monofloral Honeys

Thyme, cotton, and heather honey samples were selected for the creaming process, as these samples exhibited the most diverse compositional characteristics (Table 3), considering that sugar composition is a strong determinant for crystallization. Specifically, cotton honey samples had the highest mean glucose content, F+G sum and G/W ratio and the lowest F/G, while heather honeys showed the opposite trends. On the other hand, honeys from thyme tested in the present study exhibited an intermediate glucose content and high F/G ratio, and coincidentally high F+G, and G/W values. Honeys from cotton and thyme are known for their rapid crystallization and usually tend to crystallize quickly in the package, facing challenges during standardization and commercialization of these products. Moreover, thyme honey was chosen for its distinctive aroma, aiming to provide in the market an alternative spreadable product with improved sensorial quality.

**Figure 6:**
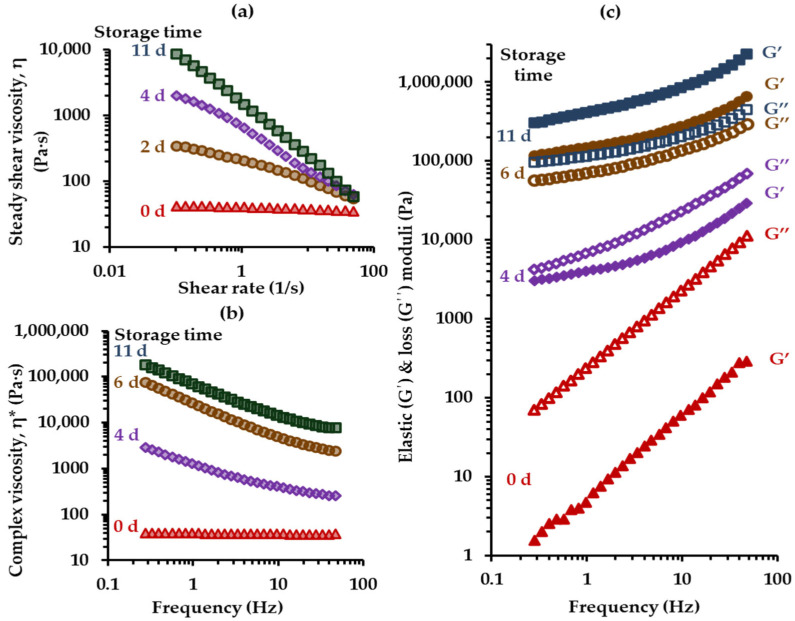
Steady shear (**a**) and oscillatory (**b**,**c**) rheological measurements for monitoring the steady shear (**a**) and complex (**b**) viscosity, and storage and loss moduli (**c**) of a representative honey sample (cotton 2) during the creaming–crystallization process performed at 14 °C; both rheological tests were carried out at 20 °C and the oscillatory measurements were made at 0.1% strain.

The representative honey samples were subjected to the creaming process by storage at 14 °C for 27 days. During storage, at different time intervals, steady shear and dynamic rheological tests performed on cured honeys revealed an increase in steady shear (η) and complex (η*) viscosity, and elastic modulus (G′) as well as a gradual change of the rheological behavior from a Newtonian-like to that of a typical pseudoplastic and viscoelastic fluid as the creaming process due to crystal formation progresses (Figure 6). At the beginning of creaming, honey exhibited Newtonian behavior, as η (Figure 6a) and η* (Figure 6b) viscosities were independent of shear rate ($\dot{\gamma \;}$) and frequency, respectively, while G′ and G″ moduli were strongly dependent on frequency and the difference between their values was large over the tested frequency range (Figure 6c).

As storage time increased, the cured samples exhibited pseudoplastic behavior, with viscosity parameters showing dependence on both shear rate and frequency (Figure 6a,b). Additionally, the dependence of G′ and G″ curves on frequency became milder and the difference between G′ and G″ curves decreased, showing a shift from Newtonian to viscoelastic behavior. With further increase in storage time, the G′ values became predominant over the G″ values, indicating the formation of a weak crystal network (Figure 6c). Over time, the structured network of the creamed honeys was further strengthened, as evidenced by the increase in difference between the G′ and G″ parameters and the increase in the steepness of the flow curves (Figure 6a); the latter is pointing out a sharp destruction of the structured crystalline network upon shear deformation.

**Figure 7:**
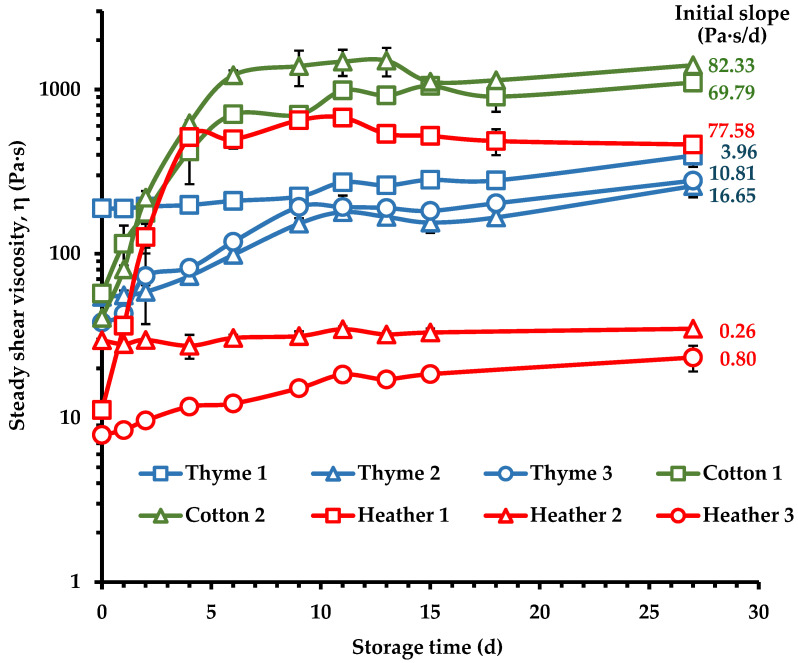
Steady shear viscosity (η) at 1 s^−1^ of creamed honey samples during the creaming process at 14 °C; calculated initial slopes of the η vs. time curve are also shown on the graphs.

**Figure 8:**
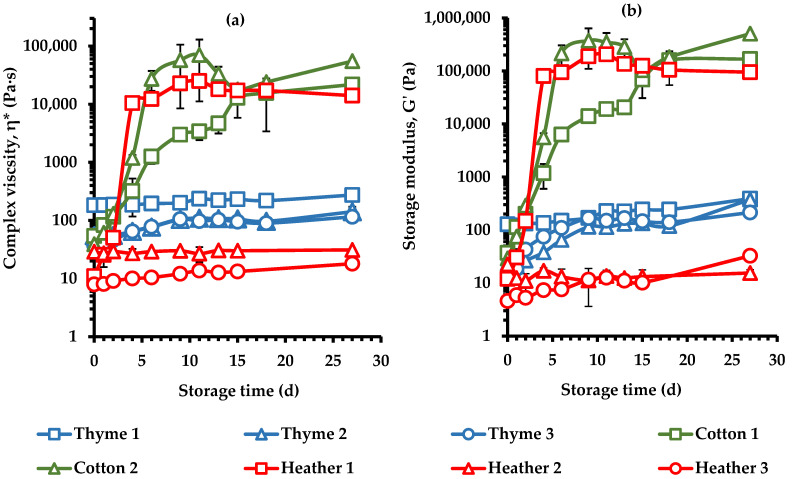
Complex viscosity, (η*) at 1 Hz (**a**) and elastic modulus (G′) at 1 Hz (**b**) of creamed honey samples during the creaming process at 14 °C for 27 d; measurements were performed at 0.1% strain.

**Figure 9:**
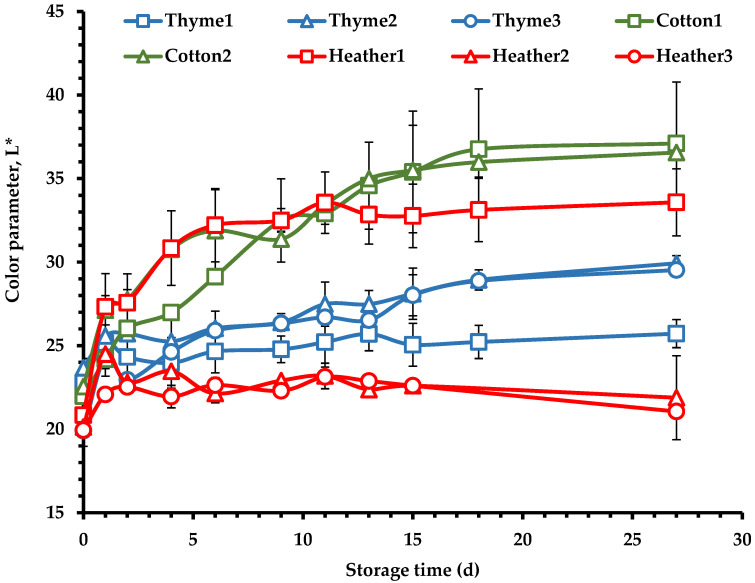
Color parameter L* of honey samples during the creaming process at 14 °C for 27 d.

The rate and degree of the crystallization process were evaluated by monitoring the changes in steady shear and dynamic rheological parameters as well as the L* color parameter of the cured honey samples as a function of time (Figure 7, Figure 8 and Figure 9). As a result of the crystallization process, the kinetics demonstrated an increase in steady shear viscosity (Figure 7), complex viscosity (Figure 8a), elastic modulus (Figure 8b), and color lightness (Figure 9) upon evolution of the creaming process.

Specifically, the rheological measurements used to monitor crystallization kinetics, indicated that cotton honey exhibited a high creaming rate, while thyme honey showed a low rate. The crystallization behavior of the heather honey samples varied, with one sample exhibiting rapid crystallization (Heather 1), whereas the other two without crystallization (Heather 2 and 3). Cotton honeys (Cotton 1, 2) and Heather 1 samples showed a significant increase in apparent viscosity (η) within the first nine days of storage, but no further changes were noted with extended storage. Moreover, these samples displayed the highest initial slopes of the evolution curve of η over storage time (Figure 7), i.e., rate of crystallization. In the case of cotton honeys, this behavior is likely attributed to the low F/G ratio (1.01 ± 0.02) and the high glucose content (38.42 ± 2.33), and G/W ratio (2.43 ± 0.16) of these samples (Table 3), which are linked with a high crystallization rate; honeys with F/G < 1.11 or/and G/W > 2.0 ratios are known to crystallize faster [17,18,75]. As was expected, the cotton honey samples, due to the above compositional characteristics, crystallized more rapidly than all the other tested samples. Fructose content, water content, and G/W ratio have been previously reported as key indicators influencing the rheological parameters of honey samples [23,55].

The crystallization rate of thyme honey remained low, while the heather honey samples exhibited a wide range of crystallization behaviors (Figure 7). Heather 1 exhibited a high crystallization rate similar to that of cotton honeys, while Heather 2 and Heather 3 showed a very slow creaming rate and low degree of crystallization (low η values throughout the storage time). This behavior is possibly attributed to the higher glucose content (37.2% *w*/*w*) and G/W (2.04) ratio, and the lower F/G (1.03) ratio of Heather 1 honey, compared to the 24.9 and 26.8% (*w*/*w*) glucose, 1.53 and 1.35 G/W, and 1.19 and 1.09 F/G ratio of Heather 2 and Heather 3 samples, respectively. All the three tested thyme honey samples, although they had a high G/W ratio (1.98–2.49), had a F/G ratio greater than 1.11, ranging from 1.18 to 1.29, which is consistent with a product of medium crystallization rate, as evidenced by their kinetic responses (Figure 7). Variations in crystallization rates based on F/G ratios have been previously documented. Dettori et al. [13], observed a pertinent relationship between these parameters, where higher F/G ratios corresponded with slower crystallization. Beyond the F/G ratio, other factors such as sucrose and water content, and G/W ratio, significantly influence the crystallization process [12].

The high initial viscosity of liquified thyme honeys (Figure 1 and Figure 2) could also contribute to this direction by decreasing the molecular mobility and diffusion; thus, the Thyme 1 sample which exhibited the lowest initial slope of η-time curve among the thyme honeys was the liquified sample with the highest η value (Figure 2). For samples undergoing crystallization upon storage, the viscosity increases, and the product solidifies, as the crystal structures are being developed [30]. These differences among the tested cotton, thyme, and heather honey samples concerning their crystallization patterns (Figure 7) were also noted for η* (Figure 8a), and G′ (Figure 8b) in the respective creaming kinetics. All these findings further point to the association between the sugar composition of honey samples and the recorded changes in the rheological properties as creaming occurs [23,77].

The shear stress data as a function of shear rate (flow curves) of the honey samples during their creaming process were successfully fitted to the power-law model (R² = 0.92–1.00). At day 0, all liquefied honey samples exhibited flow behavior index (n) values close to 1.00 (Table 4), indicative of Newtonian-like fluid behavior [78]. However, these values gradually decreased during storage and all creamed honey samples, except Heather 2 and Heather 3, behave as shear-thinning dispersions, where the viscosity decreases with the shear rate (0 < n < 1) [77]. The Cotton 2 sample showed the lowest n value following storage, most likely due to the higher concentration of crystals, reflecting a more extended network structure of crystal particles which is deformed with increasing shear rate. On the other hand, Heather 2 and Heather 3 have shown no evidence of crystallization since their n values are 0.99 and 0.96 even after 27 days of storage.

Regarding the coefficient of consistency (K values, Table 4) derived from the power-law fitting, there was an increase in all tested samples after 27 days of storage, also signaling the development of crystallization. At the end of storage period, the K value was highest for Cotton 2, followed by the Cotton 1, Heather 1, Thyme honeys, and Heather 2 and Heather 3 samples. This suggests that cotton honeys tend to crystallize to a higher extent than the other samples tested, which is also consistent with their highest η values at the end of the creaming process (Figure 7).

Colorimetric measurements also showed increases in the L* color parameter upon honey crystallization (Figure 9). After 13 days of storage, a plateau was reached, as has been also reported by other authors [13,79]. The color parameters of creamed honeys may be influenced by the size and shape of the formed crystals [80], where the increased L* values are likely due to light reflection from the formed glucose crystals [13]. Furthermore, increased L* values may be linked to the rate and extent of crystallization, as samples with higher glucose content and certain favoring crystallization indices (F/G, G/W) tend to crystallize more quickly. The colorimetric measurements also showed that cotton honey samples exhibited the highest L* values, followed by heather 1, thyme samples, and finally heather 2 and heather 3 (Figure 9), consistent with the compositional data of these samples (Table 3) and in agreement with the findings from the rheological responses of the stored honeys throughout the creaming process (Figure 7 and Figure 8).

The above trends of the experimental data were further examined by Pearson’s correlation analysis. This analysis examined the relationships among the rheological and color parameters (G, η, η*, K, n, L*) at the end of the creaming process (27th day) and the compositional characteristics of honey samples. A strong positive correlation was observed between L* parameter and glucose content (r = 0.964, *p* < 0.01), indicating that a higher glucose content is associated with increased lightness in the creamed honey products, possibly due to enhanced crystallization. Moreover, η showed a positive correlation with glucose content (r = 0.942, *p* < 0.05), suggesting that as the glucose content in honey increases, the viscosity of the creamed products also tends to rise due to increased concentration of crystalline sugar particles.

A negative correlation was noted between the F/G ratio and G′ (r = −0.846, *p* < 0.05), implying that lower F/G ratios correspond to higher G′ values, i.e., more elastic/rigid crystal network structures. The flow behavior index (n) of creamed honeys was positively correlated with F/G (r = 0.899, *p* < 0.05), and negatively correlated with both L* parameter (r = −0.959, *p* < 0.01) and glucose content (r = −0.979, *p* < 0.01), indicating that as the glucose content increases or F/G ratio decreases, the flow behavior index tends to decrease, showing a stronger pseudoplastic behavior probably due to increased levels of crystalline particles in honey dispersions. Moreover, the initial slope of η versus storage time curve, related to crystallization rate, was positively correlated with the K factor (r = 0.907, *p* < 0.05), the L* parameter (r = 0.950, *p* < 0.01), and glucose content (r = 0.949, *p* < 0.01), while negative correlations were found with F/G (r = −0.902, *p* < 0.05) and the n index (r = −0.987, *p* < 0.01). The above trends pointed out that high glucose content and low F/G ratio in honey accelerate the creaming–crystallization process, leading to a highly viscous creamed honey product with light color and enhanced pseudoplastic behavior.

## 4. Conclusions

In this study, the compositional, physicochemical, and rheological characteristics of Greek honeys from different botanical origins, as well as their potential use in forming creamed honey products, were explored. All samples exhibited low moisture content and water activity (a_w_) values, ensuring optimal chemical and microbiological stability. The botanical origin significantly influenced the color parameters, while other quality indicators (HMF levels and diastase activity) confirmed the freshness of all honey samples examined (i.e., absence of thermal pretreatment). Fructose was the predominant sugar, with cotton and thyme honeys showing the highest combined fructose+glucose content—a factor contributing to their crystallization propensity.

Rheological measurements of liquefied honeys revealed a Newtonian behavior, with viscosity following an exponential increase as the moisture content decreased. DSC analysis showed decreasing values of the glass transition temperature (Tg) of honey solids with increasing moisture content, with the Gordon–Taylor model confirming the water’s plasticizing effect on the amorphous matrix of sugars. PCA revealed clustering of honey samples in groups based on similar compositional and physicochemical parameters according to their plant origin.

Further testing of thyme, cotton, and heather honeys throughout a creaming process showed an evolution of increasing viscosity (η), complex viscosity (η*), elastic modulus (G′), and color parameter, L*, with storage time. These changes were also accompanied by a shift from a Newtonian-like to a pseudoplastic (shear-thinning) and a more solid-like rheological behavior. Power law modeling revealed a decreasing flow behavior index (n) and increasing consistency coefficient (K), indicative of a strengthened network structure with crystalline sugar particles dispersed in the liquid medium. Notably, the crystallization kinetics varied significantly with the botanical origin of the samples: cotton honey crystallized most rapidly and extensively, while thyme honey showed slower crystallization rates, resulting in weaker crystal networks. Heather honeys exhibited divergent behaviors, ranging from rapid crystallization to a complete absence of structured networks. Overall, the results demonstrate that glucose content and fructose/glucose (F/G) ratio are critical factors governing the creaming behavior, where elevated glucose content and reduced F/G ratios promote more robust crystal development and firmer final textures in the product. In conclusion, the honey crystallization rate is largely influenced by the botanical origin of the samples, and certain compositional and physicochemical parameters can effectively serve as useful quality indices in predicting the crystallization propensity of the product as well as the changes associated with the creaming process. The established relationships among sugar composition, rheological properties, and crystallization kinetics would enable targeting-selection of honey types and processing conditions for standardized creamed honey production.

## Figures and Tables

**Table 1:** Physical characteristics of Greek honey samples from six species.

Plant Origin	Moisture Content (%)	aw	Electrical Conductivity (mS/cm)	Color Parameters		
				L*	a*	b*
Thyme (n = 5)	14.60 ± 1.09 ^a 1^	0.53 ± 0.02 ^a^	0.36 ± 0.09 ^a^	33.17 ± 0.64 ^cd^	1.52 ± 0.62 ^b^	6.03 ± 0.34 ^bc^
C. thorn (n = 5)	16.08 ± 0.83 ^a^	0.57 ± 0.02 b^c^	0.89 ± 0.15 ^b^	31.88 ± 0.72 ^b^	2.74 ± 0.38 ^cd^	5.20 ± 1.03 ^b^
Chestnut (n = 4)	15.75 ± 0.57 ^a^	0.56 ± 0.01 ab^c^	1.32 ± 0.10 ^c^	30.44 ± 0.16 ^a^	2.91 ± 0.21 ^d^	2.81 ± 0.34 ^a^
Heather (n = 3)	18.10 ± 1.43 ^a^	0.60 ± 0.02 ^c^	1.25 ± 0.04 ^c^	29.50 ± 0.09 ^a^	2.34 ± 0.37 ^bcd^	1.26 ± 0.18 ^a^
Orange (n = 3)	16.43 ± 0.39 ^a^	0.57 ± 0.01 ^abc^	0.28 ± 0.01 ^a^	34.40 ± 0.58 ^cd^	0.21 ± 0.29 ^a^	5.43 ± 0.97 ^b^
Cotton (n = 2)	15.85 ± 0.35 ^a^	0.53 ± 0.00 ^bc^	0.75 ± 0.16 ^b^	35.71 ± 0.69 ^d^	1.36 ± 0.78 ^abc^	7.85 ± 0.45 ^c^

^1^ Mean values with different letters in the same column are significantly different according to Tukey’s test (*p* < 0.05).

**Table 2:** Chemical characteristics of Greek honey samples from six plant origins.

	HΜF (mg/kg)		Diastase Activity (DN)	
Plant Origin	Range ^1^	Average Value ^2^	Range ^1^	Average Value ^2^
Thyme (n = 5)	6.0–27.7	13.6 ± 8.1 ^a 3^	7.0–17.0	14.00 ± 3.8 ^a,b^
C. thorn (n = 5)	0.0–10.6	4.1 ± 3.8 ^a^	18.5–32.0	25.7 ± 5.1 ^c^
Chestnut (n = 4)	0.0–1.6	0.6 ± 0.7 ^a^	14.3–27.9	23.6 ± 5.5 ^b,c^
Heather (n = 3)	5.2–37.1	16.8 ± 14.4 ^a^	4.7–14.7	8.2 ± 4.6 ^a^
Orange (n = 3)	0.2–6.4	3.4 ± 2.6 ^a^	6.4–15.4	11.2 ± 3.7 ^a,b^
Cotton (n = 2)	3.1–9.4	6.3 ± 3.1 ^a^	14.8–14.8	14.8 ± 0.0 ^a,b,c^

^1^ Value range of honeys with the same plant origin. ^2^ Mean values of honeys from the same plant origin. ^3^ Mean values with different letters in the same column are significantly different according to Tukey’s test (*p* < 0.05).

**Table 3:** Sugar content of Greek honey samples from six species.

Plant Origin	Sugars (g/100 g)										
	Fructose	Glucose	Fructose + Glucose	Fructose/ Glucose	Glucose/ Water	Sucrose	Maltose	Trehalose	Melezitose	Melibiose	Raffinose
Thyme (n = 5)	40.75 ± 5.18 ^b 1^	31.96 ± 4.38 ^ab^	72.72 ± 4.39 ^b^	1.28 ± 0.11 ^abc^	2.30 ± 0.30 b^c^	1.52 ± 0.45 ^a^	3.13 ± 0.77 ^a^	0.48 ± 0.18 ^ab^	n.d.	2.24 ± 0.29 ^b^	0.83 ± 0.22
C. thorn (n = 5)	34.04 ± 1.66 ^ab^	24.72 ± 2.13 ^a^	58.79 ± 4.66 ^a^	1.38 ± 0.06 ^c^	1.54 ± 0.14 ^a^	n.d. ^2^	3.23 ± 1.68 ^a^	0.71 ± 0.23 ^b^	n.d.	0.08 ± 0.01 ^a^	n.d.
Chestnut (n = 4)	36.36 ± 0.90 ^ab^	27.73 ± 2.94 ^a^	64.09 ± 4.31 ^ab^	1.32 ± 0.15 ^bc^	1.76 ± 0.15 ^ab^	n.d.	1.54 ± 0.52 ^a^	0.08 ± 0.01 ^a^	n.d.	0.37 ± 0.07 ^a^	n.d.
Heather (n = 4)	32.46 ± 5.12 ^a^	29.61 ± 6.61 ^ab^	62.07 ± 1.43 ^ab^	1.11 ± 0.07 ^ab^	1.64 ± 0.29 ^a^	n.d.	2.11 ± 0.17 ^a^	0.45 ± 0.21 ^ab^	n.d.	n.d.	n.d.
Orange (n = 3)	37.67 ± 0.79 ^ab^	32.46 ± 0.44 ^ab^	70.12 ± 2.61 ^ab^	1.16 ± 0.03 ^abc^	1.98 ± 0.07 ^abc^	0.41 ± 0.12 ^a^	1.66 ± 1.20 ^a^	0.09 ± 0.02 ^ab^	n.d.	n.d.	n.d.
Cotton (n = 2)	39.01 ± 1.20 ^ab^	38.42 ± 2.33 ^b^	77.43 ± 0.30 ^b^	1.01 ± 0.02 ^a^	2.43 ± 0.16 ^c^	n.d.	1.73 ± 0.33 ^a^	0.39 ± 0.09 ^ab^	n.d.	0.15 ± 0.02 ^a^	n.d.

^1^ Mean values with different letters in the same column are significantly different according to Tukey’s test (*p* < 0.05). ^2^ n.d.: not detected.

**Table 4:** Parameters of the power-law model fitted to shear stress data as a function of shear rate (at 20 °C) of honey during the creaming process at 14 °C.

Storage Time (d)	Cotton1	Cotton2	Heather1	Heather2	Heather3	Thyme1	Thyme2	Thyme3
	Consistency Factor (K, Pa∙s^n^)							
0	55.636 ^a,D 1^	39.663 ^a,C^	11.429 ^a,A^	30.023 ^abc,B^	7.830 ^a,A^	187.270 ^a,E^	54.360 ^a,D^	38.103 ^a,C^
1	96.710 ^a,E^	69.670 ^a,D^	29.614 ^b,B^	28.326 ^a,B^	7.767 ^a,A^	185.980 ^a,F^	54.776 ^a,CD^	41.716 ^a,BC^
2	148.815 ^a,C^	175.795 ^ab,C^	112.925 ^c,B^	28.361 ^ab,AB^	9.458 ^ab,A^	191.775 ^a,C^	57.937 ^a,AB^	54.567 ^a,AB^
4	345.190 ^b,C^	542.195 ^b,D^	544.409 ^d,D^	27.888 ^a,A^	10.916 ^b,A^	194.39 ^a,B^	68.908 ^ab,A^	78.597 ^b,A^
6	611.095 ^c,C^	1144.800 ^c,D^	528.585 ^d,C^	29.424 ^abc,A^	11.222 ^bc,A^	204.285 ^a,B^	86.107 ^b,A^	97.227 ^c,AB^
9	732.110 ^cd,C^	1420.850 ^cd,D^	692.175 ^d,C^	31.293 ^bcd,A^	14.172 ^cd,A^	212.215 ^ab,B^	124.710 ^c,B^	147.535 ^d,B^
11	910.815 ^de,C^	1477.150 ^d,D^	724.100 ^c,C^	33.668 ^a,A^	16.647 ^cd,A^	248.140 ^c,B^	146.505 ^c,B^	151.835 ^d,B^
13	752.085 ^cd,D^	1287.550 ^cd,E^	566.300 ^b,C^	31.599 ^cd,A^	15.512 ^c,A^	242.075 ^bc,B^	145.630 ^c,B^	150.625 ^d,B^
15	1000.555 ^e,D^	1021.600 ^c,D^	552.140 ^b,C^	32.027 ^cd,A^	17.020 ^de,A^	257.275 ^c,B^	134.250 ^c,B^	148.531 ^d,B^
18	844.890 ^cde,D^	1053.600 ^cd,E^	501.730 ^c,C^	33.077 ^d,A^	19.138 ^ef,A^	253.750 ^c,B^	139.080 ^c,B^	155.82 ^d,B^
27	1014.915 ^e,E^	1336.700 ^cd,F^	466.905 ^b,D^	34.011 ^d,A^	21.103 ^f,A^	338.140 ^d,C^	205.300 ^d,B^	209.260 ^e,B^
	**Flow behavior index (n)**							
0	0.983 ^d,B^	0.986 ^h,B^	0.964 ^f,A^	0.989 ^ab,B^	0.996 ^bcd,B^	0.991 ^d,B^	0.996 ^d,B^	0.992 ^f,B^
1	0.931 ^d,A^	0.943 ^h,A^	0.908 ^f,AB^	0.988 ^ab,C^	1.000 ^d,C^	0.993 ^d,C^	0.997 ^d,C^	0.988 ^ef,BC^
2	0.878 ^d,B^	0.809 ^g,A^	0.775 ^e,A^	0.983 ^ab,C^	0.986 ^bc,C^	0.991 ^d,C^	0.990 ^d,C^	0.938 ^de,BC^
4	0.698 ^c,C^	0.505 ^f,B^	0.225 ^ab,A^	0.979 ^a,E^	0.9886 ^bc,E^	0.991 ^d,E^	0.988 ^d,E^	0.874 ^ab,D^
6	0.537 ^b,C^	0.275 ^bc,B^	0.224 ^ab,A^	0.999 ^b,E^	0.977 ^abc,E^	0.988 ^d,E^	0.972 ^cd,E^	0.922 ^cd,D^
9	0.477 ^ab,B^	0.217 ^a,A^	0.192 ^a,A^	0.982 ^ab,D^	0.980 ^abc,D^	0.982 ^cd,D^	0.942 ^bc,D^	0.877 ^bc,C^
11	0.391 ^ab,B^	0.276 ^bc,A^	0.224 ^ab,A^	0.993 ^ab,D^	0.993 ^bcd,D^	0.979 ^cd,D^	0.930 ^b,CD^	0.879 ^bc,C^
13	0.474 ^a,C^	0.313 ^cd,B^	0.260 ^bc,A^	0.990 ^ab,F^	1.000 ^cd,F^	0.971 ^c,EF^	0.932 ^b,DE^	0.899 ^bcd,D^
15	0.384 ^a,B^	0.375 ^e,B^	0.293 ^cd,A^	0.988 ^ab,E^	0.982 ^abc,E^	0.970 ^c,E^	0.929 ^b,D^	0.894 ^bcd,C^
18	0.376 ^a,A^	0.340 ^de,A^	0.313 ^cd,A^	0.990 ^ab,C^	0.972 ^ab,C^	0.947 ^a,BC^	0.930 ^b,BC^	0.886 ^bcd,B^
27	0.365 ^a,B^	0.246 ^ab,A^	0.328 ^d,B^	0.995 ^ab,F^	0.955 ^a,EF^	0.906 ^a,DE^	0.857 ^a,CD^	0.821 ^a,C^

^1^ Mean values with different lowercase letters in the same column are significantly different and mean values with different uppercase letters in the same row are significantly different (Tukey’s test, *p* < 0.05).

## Data Availability

The original contributions presented in the study are included in the article; further inquiries can be directed to the corresponding author.
